# Brivaracetam exhibits mild pro-inflammatory features in an *in vitro* astrocyte-microglia co-culture model of inflammation

**DOI:** 10.3389/fncel.2022.995861

**Published:** 2022-11-03

**Authors:** Fatme Seval Ismail, Pedro M. Faustmann, Marie-Luise Kümmel, Eckart Förster, Timo Jendrik Faustmann, Franco Corvace

**Affiliations:** ^1^Department of Neurology, University Hospital Knappschaftskrankenhaus Bochum, Ruhr University Bochum, Bochum, Germany; ^2^Department of Neuroanatomy and Molecular Brain Research, Ruhr University Bochum, Bochum, Germany; ^3^Department of Psychiatry and Psychotherapy, Medical Faculty, Heinrich Heine University, Düsseldorf, Germany

**Keywords:** brivaracetam, astrocyte-microglia co-culture model, inflammation, connexin 43, gap junctions, toxicity

## Abstract

Implications of glia in the pathophysiology of epilepsy raise the question of how these cells besides neurons are responsive to antiseizure medications (ASMs). Understanding ASM effects on glia and glia-mediated inflammation may help to explore astrocytes and microglia as potential targets for alternative anti-epileptogenic therapies. The aim of this study was to investigate the effects of the new generation ASM brivaracetam (BRV) in an astrocyte-microglia co-culture model of inflammation. Primary rat astrocytes co-cultures containing 5%–10% (M5, “physiological” conditions) or 30%–40% (M30, “pathological inflammatory” conditions) of microglia were treated with different concentrations of BRV (0.5, 2, 10, and 20 μg/ml) for 24 h. Glial cell viability was measured by MTT assay. Microglial activation states were analyzed by immunocytochemistry and astroglial connexin 43 (Cx43) expression by Western blot analysis and immunocytochemistry. Gap-junctional coupling was studied *via* Scrape Loading. Incubation with high, overdose concentration (20 μg/ml) of BRV significantly reduced the glial cell viability under physiological conditions (*p* < 0.01: **). Treatment with BRV in therapeutic concentrations (0.5 and 2 μg/ml) reduced the resting microglia (*p* < 0.05: *) and increased the microglial activation under inflammatory conditions (*p* < 0.01: **). Astroglial Cx43 expression was not affected. The gap-junctional coupling significantly increased only by 0.5 μg/ml BRV under physiological conditions (*p* < 0.05: *). Our findings suggest mild pro-inflammatory, *in vitro* features of BRV with regard to microglia morphology. BRV showed no effects on Cx43 expression and only limited effects on gap-junctional coupling. Reduction of glial viability by overdose BRV indicates possible toxic effects.

## Introduction

Epilepsy is a chronic neurological disorder characterized by a predisposition to generate recurrent epileptic seizures due to abnormal excessive or synchronous neuronal activity in the brain (Fisher et al., 2005). The pooled lifetime prevalence of epilepsy was reported by 7.60 per 1,000 persons (95% CI 6.17–9.38; Fiest et al., 2017). It is estimated that more than 50 million people worldwide suffer from epilepsy, so it is one of the most common neurological disorders, affecting people of all ages (Vezzani et al., 2011; Patel et al., 2019). Despite a wide range of available antiseizure medications (ASMs), about 30% of patients with epilepsy are refractory to ASMs (Perucca et al., 2007). As a consequence, epilepsy leads to social and economic impacts (Fisher et al., 2005; Beecham et al., 2010). Antiseizure medications targeting neuronal cells form the most common pharmacologic therapy approach (Asconapé, 2010) and an early response to treatment predicts a good long-term prognosis (Beghi et al., 2015). There is strong evidence of glial involvement in the pathophysiology of epilepsy (Patel et al., 2019). Glial cells represent a population of non-neuronal cells of the central nervous system (CNS) including astrocytes and microglia. Astrocytes are the main glial cell population and are involved in the stabilization of the neuronal network, supporting the blood-brain barrier, and maintaining brain homeostasis. Various glial mechanisms have been described that contribute to ictogenesis and epileptogenesis e.g., differential regulation of astrocytic gap junctional (GJ) protein connexin 43 (Cx43; Giaume et al., 2010; Patel et al., 2019). Connexins form hexamers, termed connexons, which are embedded in the plasma membrane. When connexons are localized head-to-head between two neighboring cells, they can dock to form a GJ channel (GJC). These GJCs allow direct cell-to-cell communication, whereas Cx hemichannels contribute to the exchange of ions and signaling molecules between the cytoplasmic and extracellular milieu (Dermietzel et al., 1991; Giaume et al., 1991, 2010; Nagy et al., 1999). Cx43 and Cx30 are the main connexins expressed in astrocytes (Dermietzel et al., 1991; Giaume et al., 1991, 2010; Nagy et al., 1999). Previous studies suggest that Cx-based GJs and hemichannels are involved in epilepsy. In addition to interneuronal GJ communication, inter-glial GJs are also considered to contribute to seizure generation (Szente et al., 2002; Condorelli et al., 2003; Samoilova et al., 2003; Mylvaganam et al., 2010, 2014). An upregulation of glial, but not neuronal gap junctional mRNA and protein was observed in most animal seizure models and human patients with epilepsy, e.g., increased expression of astrocytic Cx30 and Cx43 mRNAs compared to neuronal Cx mRNAs in mammalian hippocampal and cortical tissue (Condorelli et al., 2003; Samoilova et al., 2003; Mylvaganam et al., 2014). In addition, the astrocytic Cx43 expression was significantly upregulated in an *in vitro* seizure model, supporting the role of glia in ictogenesis (Mylvaganam et al., 2010, 2014). Moreover, gliosis and uncontrolled inflammation were described during epileptogenesis (Vezzani et al., 2011; Sofroniew, 2014; Patel et al., 2019). Gliosis is associated with physicochemical and physiological changes in glial cells, particularly astrocytes and microglia, as response to diverse CNS diseases (Sofroniew, 2014). The main features of gliosis are hypertrophy of the cells, upregulated expression of several proteins in astrocytes, and ionized calcium-binding adaptor molecule 1 (IBA1) and CD68 in microglia as well as cellular proliferation (Sofroniew, 2014; Patel et al., 2019). Following this, further research on specific functions and effects of astrocytes and astrogliosis may be interesting for novel therapeutic strategies for epilepsy.

Microglia, the main immune cell population in the CNS, play an important role in a healthy CNS as well as during various CNS diseases (Eyo et al., 2017). An accumulating evidence implicates morphological and molecular activation of microglia in epilepsy (Eyo et al., 2014, 2017). Furthermore, the involvement of microglia in the regulation of neuronal activities in healthy and epileptic brain is known (Pascual et al., 2012; Eyo et al., 2014, 2017). Another example of the association of chronic brain inflammation with the activation of microglia, astrocytes, and peripheral immune cells, producing inflammatory mediators, and epilepsy is immune-mediated encephalitis such as Rasmussen encephalitis (Varadkar et al., 2014). Also, different forms of autoimmune encephalitis and limbic encephalitis lead to a high incidence of seizures and confirm the involvement of immune and inflammatory mechanisms in some forms of epileptogenesis (Spatola and Dalmau, 2017; Geis et al., 2019).

In the healthy CNS, microglia typically exist in a resting ramified form, ranging from 5% to 20% of the glial cell population (Faustmann et al., 2003). Microglia are activated by various pathological events in the CNS and the microglial phenotype can change from the inactive, resting ramified type (RRT) *via* an intermediate type (INT) to the activated, round phagocytic type (RPT; Faustmann et al., 2003; Block et al., 2007; Wolf et al., 2017). Faustmann et al. (2003) developed an *in vitro* astrocyte-microglia co-culture model, allowing to study the physiological as well as pathological inflammatory conditions in the CNS depending on the percentage and activation of microglia (Faustmann et al., 2003). This *in vitro* model has been proven as suitable for the investigation of pharmacological effects on neuroinflammation (Ismail et al., 2021). Different ASMs such as levetiracetam (LEV), valproic acid (VPA), gabapentin (GBT), phenytoin (PHE), carbamazepine (CBZ), lacosamide (LCM), lamotrigine (LTG), and topiramate (TPM) have already been studied in our co-culture model with regard to glia-mediated pro- and anti-inflammatory effects (Haghikia et al., 2008; Stienen et al., 2011; Dambach et al., 2014; Ismail and Faustmann, 2021; Ismail et al., 2021; Corvace et al., 2022; Faustmann et al., 2022).

To sum up, all these findings support the implication of astrocyte dysfunction and glia-mediated inflammation in epilepsy. Astrocytes and microglia can serve as potential novel targets for alternative anti-epileptogenic therapies in the future.

Brivaracetam (BRV) (2S)-2-[(4R)-2-oxo-4-propylpyrrolidinyl] butanamide is a new generation ASM (available orally and intravenously) approved for use as adjunctive therapy of focal-onset seizures with or without secondary generalization in adults, adolescents and children aged >2 years with epilepsy. Brivaracetam is similar to LEV a member of the racetam class of ASMs whose antiepileptic mechanism encompasses a selective binding to the synaptic vesicle protein type 2A (SV2A), modulating the presynaptic neurotransmitter release with inhibition of neuronal hyperexcitability (Klitgaard et al., 2016; Wood and Gillard, 2017). However, BRV is characterized by higher affinity and differential interaction with SV2A compared to LEV as well as higher lipophilicity and more rapid brain penetration, leading to more potent anti-epileptogenic features (Klitgaard et al., 2016).

The involvement of astrocytes and microglia in the pathophysiology of epilepsy raises the question of how these cells besides neurons may be responsive to current ASMs. Little is known about the direct effects of the new generation ASM BRV on glial cells (Tsymbalyuk et al., 2019; Okada et al., 2021). Therefore, we aimed to study the concentration-dependent effects of BRV on glial viability, microglial activation, expression of GJ protein Cx43 as well as intercellular communication in an *in vitro* astrocyte-microglia co-culture model of inflammation.

## Materials and Methods

### Cell culture

The preparation of primary astrocyte-microglia co-cultures was carried out according to the protocol of Faustmann et al. (2003) using brain hemispheres of postnatal Wistar rats (postnatal day 0–2, P0–P2). All experiments complied with the ethical standards of Ruhr University Bochum and the ARRIVE guidelines, and were in accordance with the German Animal Welfare Act. The experiments were approved by the local authorities in Bochum, Germany. Animals had free access to water and food under standard laboratory conditions. The P0–P2 rats were decapitated without sedation according to the German animal welfare act. Following this, the meninges and choroid plexus were removed and the brain tissue was collected in ice-cooled phosphate-buffered saline (PBS; containing 1.38 M NaCl, 27 mM KCl, 81 mM NaH_2_PO_4_, 14.7 mm K_2_H_2_PO_4_; J.T. Baker, Deventer, The Netherlands). The collected brain tissue was treated with 0.1% trypsin solution at 37°C for 30 min (PAA laboratories, Pasching, Austria) and centrifugated at 500× *g* for 12 min. After removal of the supernatant, the pellet was resuspended in 5 ml DNase I solution (Serva Electrophoresis, Heidelberg, Germany; 100 μl/ml with Dulbecco’s minimal essential medium, DMEM, Invitrogen, Karlsruhe, Germany) and incubated for 5 min at room temperature. The cell suspension was filled up to a total volume of 20 ml with wash medium (containing 10% fetal calf serum (FCS; Biochrom AG, Berlin, Germany) and was centrifuged at 200× *g* for 5 min. The wash medium was removed and the pellet was resuspended in 2 ml DMEM per brain and filtered through a nylon mesh (60 μm). Cells were cultured at a density of two brains per plastic tissue-culture flask in 5% CO_2_ at 37°C in astrocyte culture medium (containing 10% FCS, 1% non-essential amino acids, 1% glutamine, 1% penicilin/streptomycin solution; PAA Laboratories, Linz, Austria). On the next day, the adherent cells were washed again and a new cell medium was added. After 5–7 days, the cells were about 100% confluent and could be passaged and used for further experiments. Depending on the extent of shaking, microglial amounts could be determined after immunocytochemical staining.

### Treatment of cultures

Based on previous studies with BRV and measured serum/plasma concentrations in patients with epilepsy (Sargentini-Maier et al., 2007; Rolan et al., 2008; Patsalos et al., 2018; Aicua-Rapun et al., 2019), the primary rat astrocytes-microglia co-cultures (M5 contains on average 5%–10% microglia and represents “physiological” conditions; M30 contains on average 30%–40% microglia and represents “pathological, inflammatory” conditions) were incubated with different concentrations of BRV (0.5, 2, 10, and 20 μg/ml; UCB Pharma S.A., Brussels, Belgium) for 24 h in 5% CO_2_ at 37°C. The high concentrations with 10 and 20 μg/ml were selected to study the toxic effects of BRV overdose (Sargentini-Maier et al., 2007; Rolan et al., 2008; Patsalos et al., 2018; Aicua-Rapun et al., 2019). Data about CSF or brain tissue concentrations of BRV in patients with epilepsy are not available. The control co-cultures were untreated with any substance/vehicle or were incubated with the vehicle 0, 9% NaCl and distilled H_2_O (at a ratio of 1:1; 2 μl per ml cell culture medium).

### MTT assay

An MTT assay (3-(4,5-dimethylthiazol-2-yl)-2, 5-diphenyltetrazolium bromide; Roche applied sciences, Penzberg, Germany) was used to detect the effects of BRV on cell toxicity, cell proliferation and viability in astrocyte-microglia co-cultures. The 96-well plates were seeded with 20,000 cells per well. After the cells were confluent, they were incubated with different concentrations of BRV as described above. Following this, 10 μl of MTT reagent was added per well and incubated for 4 h at 37°C and 5% CO_2_. As a next step, the samples were incubated with 100 μl of solubilization solution overnight. On the following day, the absorbance was determined photometrically at a wavelength of 550 nm using Bio-Rad microplate reader (München, Germany).

### Immunocytochemistry

**Figure 1:**
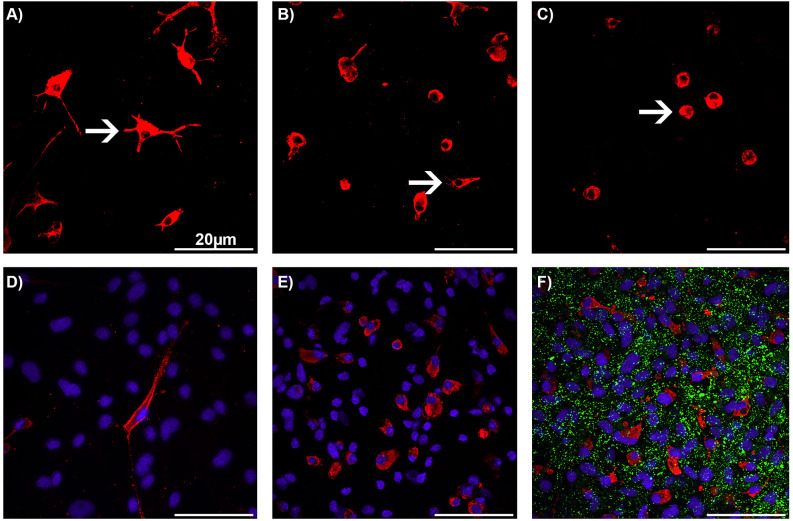
Immunocytochemistry of microglia morphology in astrocyte-microglia co-cultures after treatment with brivaracetam. Staining with the monoclonal antibody ED1 (red) allowed the classification of microglia (white arrows) as resting ramified **(A)**, intermediate **(B)**, and activated rounded phagocytic **(C)** phenotype at a magnification of 600×. Nuclei (blue) were counterstained with DAPI for visualization of the total glial cell number. A merged, representative image of physiological M5 co-cultures with a resting ramified type (RRT) of microglia **(D)** and pathological inflammatory M30 co-cultures with mainly rounded phagocytic type of microglia **(E)**. A merged image of astrocyte-microglia co-cultures after staining with the anti-Connexin (Cx) 43 antibody (green; **F**). Bars indicate 20 μm.

The microglial phenotypes and the cell numbers were analyzed using immunocytochemistry. As the astrocyte-microglia co-culture model was developed, the microglia were stained by using multiple antibody markers such as ED1, OX-42, and Isolectin B4 (IB4; Faustmann et al., 2003). The ED1 antibody showed the most intense immunoreactivity among the tested markers and allowed the classification of all microglial phenotypes as resting ramified (RRT), intermediate (INT), and activated rounded phagocytic (RPT) phenotype (Figures 1A–C). Thus, the ED1 antibody was used in further experiments with the astrocyte-microglia co-culture model for visualization of microglial phenotypes/morphology. The astrocyte-microglia co-cultures were seeded on poly-L-lysine-coated glass cover slips at 70,000 cells per well in 24-well plates and incubated with BRV for 24 h as described above. Following this, cover slips were washed with PBS and fixed with 100% ice-cooled ethanol for 10 min. The cells were washed again three times with PBS and blocked with PBS-blocking solution containing 1% bovine serum albumin (1% BSA) and 10% horse serum (HS; PAA Laboratories, Linz, Austria) for 1 h to avoid non-specific binding. The primary monoclonal antibody (mouse) anti-ED1 (anti-CD68; 1:250; Bio-Rad Laboratories, Feldkirchen, Germany) and the primary monoclonal antibody (rabbit) anti-Cx43 (1:1,000; Invitrogen, Karlsruhe, Germany) were prepared in PBS-blocking solution (PAA Laboratories, Linz, Austria). Incubation with the primary antibodies (25 μl per well) was performed in a humidified chamber at 4°C overnight. The next day, the 24-well plate was washed three times with PBS and 1% BSA. Incubation with the secondary antibodies [Alexa fluor^®^ 568 (mouse; 1:500) and Alexa fluor^®^ 488 [(rabbit; 1:500; Invitrogen, Karlsruhe, Germany) in PBS-blocking solution (25 μl per well)] was carried out for 1 h. The well plate was then washed three times with PBS for 10 min each time. Immunocytochemically labeled cells were counterstained with the nuclear dye DAPI (4’,6-diamidine-2-phenylindole; 1:1,000; Invitrogen, Karlsruhe, Germany) to quantify the cell numbers. The ratio of microglia to astrocytes was determined by comparison of the ED1-stained microglia with the total number of DAPI-labeled cells. Evaluation of the microglia morphology/phenotypes and Cx43 expression was performed in a minimum of three different visual fields on each cover slip at a primary magnification of 600× using Zeiss Axiovert 100M laser scanning confocal microscope (Carl Zeiss, Jena, Germany). The microglial phenotype RRT was distinguished by small cell bodies with a small cytoplasmic rim, and thin branching processes which are longer than the diameter of the cell body (Figure 1A); the INT had some thick pseudopodia longer than the diameter of the cell body, and a few vesicles and vacuoles in the cytoplasmic rim (Figure 1B). Rare short processes and several cytoplasmic vacuoles were typical features of activated RPT (Figure 1C). In physiological M5 co-cultures, the resting RRT microglia phenotype was dominant (Figure 1D), whereas in pathological M30 co-cultures, an increased amount of activated RPT phenotype was found (Figure 1E). The Cx43 expression varied depending on microglia activation status among the different conditions as described by Faustmann et al. (2003) (Figure 1F). Immunocytochemical Cx43 signal per cell was calculated using the ImageJ software program (Rasband, W.S. ImageJ. National Institutes of Health, Bethesda, MD, USA).

### Immunoblot (Western blot) analysis

An immunoblot (Western blot) analysis was performed to quantify all forms of Cx43 (panCx43) expression. A total number of 200,000 cells were seeded on each Petri dish (35 × 10 mm) and treated with BRV as described above. Washing with PBS and lysis with 200 μl Laemmli (1×) buffer and 4 μl protease inhibitor were performed and the cells were detached from the culture dishes using a silicone cell scraper. The lysates were stored on ice. The protein concentrations were measured by Bradford assay (Bio-Rad Bradford Protein Assay, München, Germany) according to the protocol. After loading onto 10% sodium dodecyl sulfate (SDS) gel (AppliChem, Darmstadt, Germany), electrophoresis was performed at 100 V for 20 min following 150 V for 1 h. The gels were transferred to nitrocellulose membrane and blocked with Odyssey blocking buffer (LI-COR Bioscience, Bad Homburg, Germany) for 1 h. Following this, the membranes were incubated with the primary antibodies [anti-β-actin (1:10,000; Sigma, St. Louis, USA); anti-Cx43 (1:5,000; Invitrogen, Karlsruhe, Germany)] diluted in 0.5% blocking buffer at 4°C overnight. A washing step with PBS-T [containing PBS and Tween^®^20 (Applichem, Darmstadt, Germany)] for 3 × 15 min was performed and the membranes were incubated with the secondary antibodies [IRDye 680 anti-mouse IgG (1:10,000); IRDye 800 anti-rabbit IgG (1:20,000; Invitrogen, Karlsruhe, Germany)] for 1 h. The washing step with PBS-T was repeated for 3 × 15 min and with PBS for 1 × 10 min. The bands were visualized using the LI-COR Odyssey scanner and the associated ImageStudio Lite V5.2 software (LI-COR Bioscience, Bad Homburg, Germany) was used for subsequent quantification of the bands. The percentage of Cx43 was determined using Microsoft Excel in ratio to the β-actin band.

### Scrape loading and dye transfer

The scrape loading dye transfer method (as described by El-Fouly et al., 1987) was used to investigate the effects of BRV on GJ intercellular communication and functional coupling of cells. When an injury of the cell membrane is generated, the fluorescent dye Lucifer Yellow (LY) can diffuse into adjacent cells if they are coupled by GJCs. When the cell membrane is intact and not injured, LY cannot diffuse into the cells. The cells were seeded on 24-well plates (70,000 cells per well) and incubated with BRV for 24 h as above described. After washing with PBS for 2 × 1 min, 400 μl of 0.05% (w/v) Lucifer Yellow CH solution (in PBS; Thermo Fisher Scientific, Dreieich, Germany) were added per well. The incision on the confluent cell surface was made as straight as possible from left to right with an injection cannula (0.45 × 12 mm). After incubation with LY for 2 min at 37°C protected from light, the wells were again washed with PBS for 2 × 1 min and then evaluated directly by microscopy. The functional coupling was evaluated using the Zeiss Axiovert 100M laser scanning confocal microscope (Carl Zeiss, Jena, Germany) at 10× magnification and a wavelength of 488 nm.

### Data analyses and statistics

All statistical analyses were performed with GraphPad Prism 7.0 for Windows (GraphPad Software, San Diego, USA). The normality of data distribution was analyzed with the D’Agostino-Pearson omnibus tests. Parametric tests were used when normality was given. Comparisons between more than two groups with normal distribution were analyzed using One-way analysis of variance (One-way ANOVA) followed by Kruskal-Wallis test or Bonferroni *post hoc* comparison test. The significance was set at *p* < 0.05 and the results were presented as mean ± standard error of the mean.

## Results

### Effects of BRV on glial cell viability

**Figure 2:**
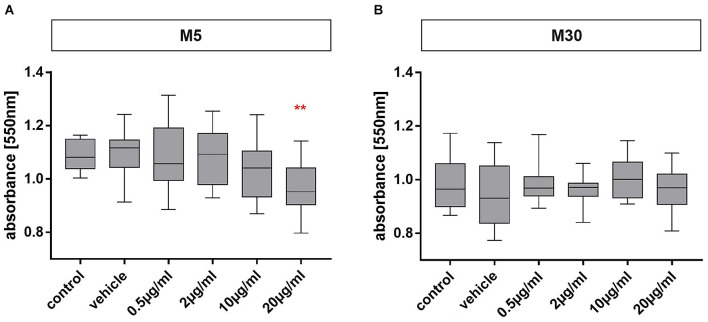
MTT assay for detection of glial viability in physiological (M5; **A**) and pathological (M30; **B**) astrocyte-microglia co-cultures after concentration-dependent incubation with brivaracetam (0.5, 2, 10, and 20 μg/ml) for 24 h. Brivaracetam significantly reduced the glial cell viability in M5 co-cultures after incubation with 20 μg/ml (*n* = 16). In M30 co-cultures, no significant changes were detected under all conditions. Comparisons between the groups were analyzed using D’Agostino-Pearson normality test and One-way analysis of variance (One-way ANOVA) followed by Bonferroni *post hoc* comparison test. Differences were considered significant at *p* < 0.01: **. Control: untreated cells with any substance/vehicle; Vehicle: cells incubated with the vehicle 0.9% NaCl diluted in distilled H_2_O (at a ratio of 1:1; 2 μl per ml cell culture medium).

Physiological M5 and pathological M30 co-cultures were incubated with 0.5, 2, 10, and 20 μg/ml of BRV for 24 h. A mean absorbance of 1.09 was detected in the physiological M5 co-cultures (Figure 2A), and the value in the pathological M30 co-cultures was 0.98 (Figure 2B). Cell viability was significant decreased after incubation with 20 μg/ml (mean absorbance of 0.96 ± 0.02; *n* = 16; *p* < 0.01: **) BRV in M5 co-cultures compared to both controls [untreated cells as control (mean absorbance of 1.09 ± 0.01; *n* = 16) and cells incubated with NaCl 0.9% diluted in H_2_O (at a ratio of 1:1) as vehicle (mean absorbance of 1.10 ± 0.02; *n* = 16)] and lower, therapeutic concentrations (0.5 and 2 μg/ml) of BRV (mean absorbance of 1.09 ± 0.03 and 1.08 ± 0.03; *n* = 16; Figure 2A). However, incubation of M30 co-cultures did not result in any significant changes (*n* = 16; Figure 2B).

### Effects of BRV on glial cell numbers and microglial activation under physiological and pathological conditions

**Figure 3:**
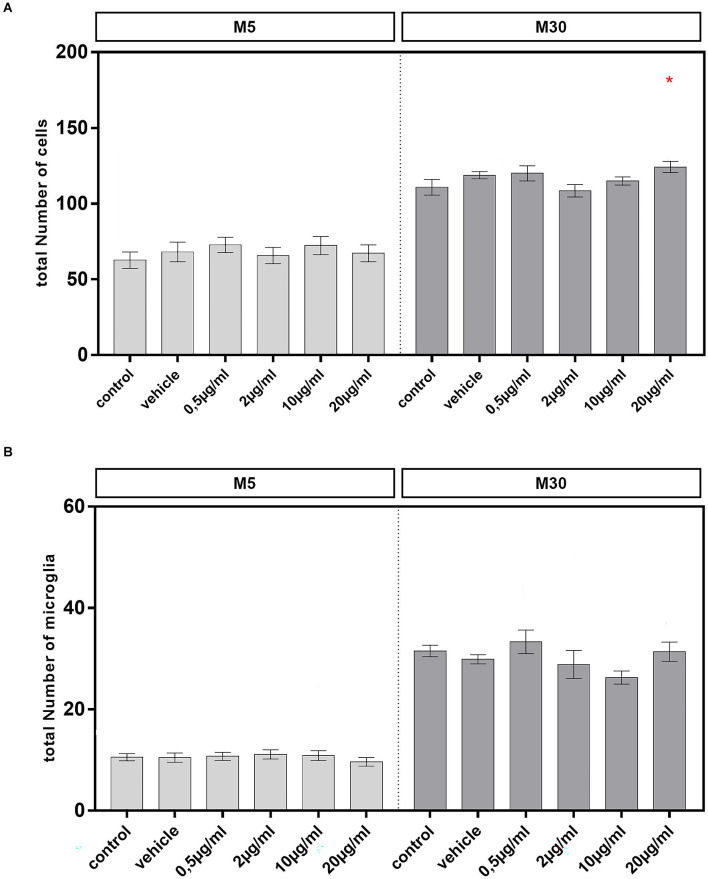
A total number of glial cells and microglial number were visualized by immunocytochemistry in physiological (M5; **A,B**) and pathological (M30; **A,B**) astrocyte-microglia co-cultures after incubation with 0.5, 2, 10, and 20 μg/ml of brivaracetam for 24 h. The total number of glial cells was significantly increased after incubation of M30 co-cultures with 20 μg/ml brivaracetam (*n* = 15). The total number of microglia was unchanged under all conditions. Comparisons between the groups were analyzed using D’Agostino-Pearson normality test and One-way analysis of variance (One-way ANOVA) followed by Bonferroni *post hoc* comparison test. Differences were considered significant at *p* < 0.05: *. Control: untreated cells; Vehicle: cells incubated with the vehicle 0.9% NaCl diluted in distilled H_2_O (at a ratio of 1:1; 2 μl per ml cell culture medium).

Incubation of pathological, inflammatory M30 co-cultures with 20 μg/ml BRV for 24 h resulted in a significant increase of the total amount of glial cells compared to untreated controls from mean 110.8% ± 5.06 in control (*n* = 15) to 124.2% ± 3.7 after incubation with 20 μg/ml BRV (*n* = 15; *p* < 0.05: *; Figure 3A). However, the total amount of glial cells in M5 co-cultures was not affected by BRV (Figure 3A). The total amount of microglia in M5 and M30 co-cultures was also unchanged (Figure 3B).

**Figure 4:**
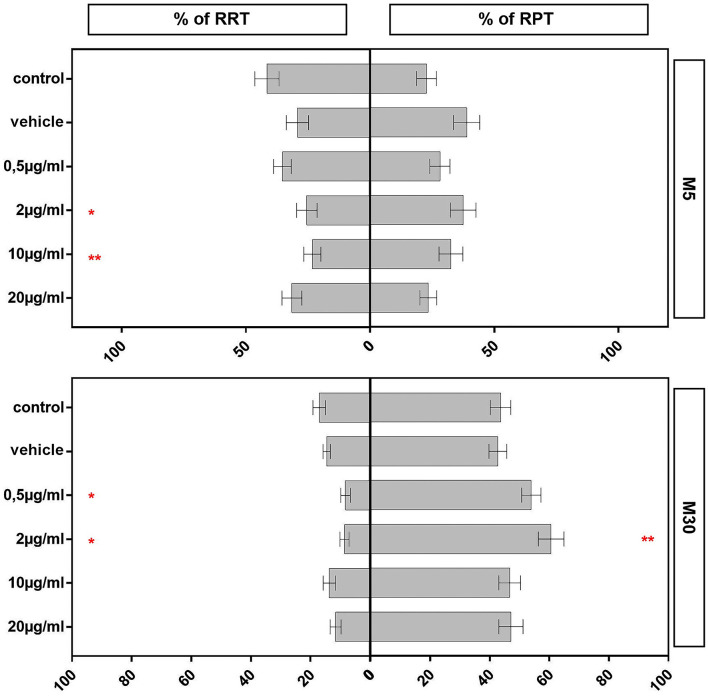
Immunocytochemical analyses of microglial phenotypes as resting ramified type (RRT) and rounded phagocytic type (RPT) in physiological M5 and pathological M30 co-cultures after concentration-dependent incubation with 0.5, 2, 10, and 20 μg/ml brivaracetam for 24 h. Incubation with brivaracetam (2 μg/ml, *n* = 35 and 10 μg/ml, *n* = 37) resulted in a significant reduction of the resting ramified type of microglia in M5 co-cultures. Further, incubation with brivaracetam at therapeutic concentrations (0.5 μg/ml, *n* = 13 and 2 μg/ml, *n* = 14) resulted in a significant reduction of the resting RRT microglia in M30 co-cultures. In parallel, the proportion of the rounded phagocytic RPT microglia significantly increased after incubation with 2 μg/ml brivaracetam (*n* = 14). Comparisons between the groups were analyzed using D’Agostino-Pearson normality test and One-way analysis of variance (One-way ANOVA) followed by Bonferroni *post hoc* comparison test. Differences were considered significant at *p* < 0.05: *, *p* < 0.01: **. Control: untreated cells; Vehicle: cells incubated with the vehicle 0.9% NaCl diluted in distilled H_2_O (at a ratio of 1:1; 2 μl per ml cell culture medium).

Incubation of physiological M5 co-cultures with 2 (*n* = 35) and 10 (*n* = 37) μg/ml BRV for 24 h resulted in a significant reduction of the resting RRT microglia compared to untreated controls (*n* = 33; from mean 41.52% ± 4.88 in control to 25.45% ± 4.15 after incubation with 2 μg/ml (*p* < 0.05: *) and to 23.17% ± 3.44 after incubation with 10 μg/ml BRV (*p* < 0.01: **; Figure 4). Under the other conditions, the microglial phenotypes were not changed in the physiological co-cultures. In M30 co-cultures, the resting RRT microglia was decreased significantly after incubation with 0.5 μg/ml (*n* = 13; *p* < 0.05: *) and 2 μg/ml (*n* = 14; *p* < 0.05: *) BRV for 24 h compared to untreated controls (*n* = 15; from mean 17.03% ± 2.13 in control to 8.19% ± 1.62 after incubation with 0.5 μg/ml and to 8.55% ± 1.52 after incubation with 2 μg/ml BRV). Consisting with this, the number of the rounded phagocytic RPT microglia was increased significantly after incubation with 2 μg/ml BRV (*n* = 13; *p* < 0.01: **) in M30 co-cultures compared to untreated controls (*n* = 15; from mean 43.65% ± 3.46 in control to 60.6% ± 4.32 after incubation with 2 μg/ml BRV; Figure 4).

### Effects of BRV on Cx43 expression in physiological and pathological astrocyte-microglia co-cultures

**Figure 5:**
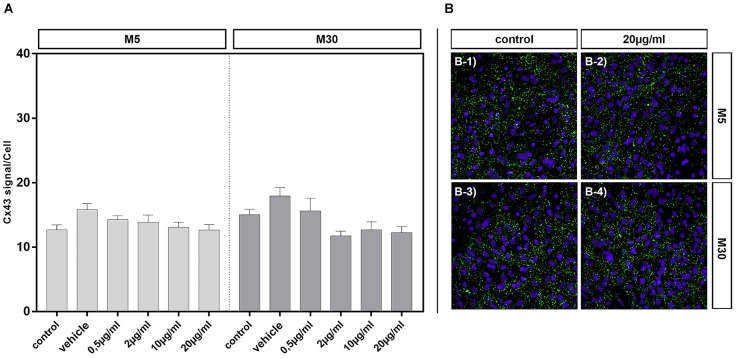
Connexin 43 (Cx43) signal per cell was detected by immunocytochemistry after concentration-dependent incubation with 0.5 (*n* = 37), 2 (*n* = 35), 10 (*n* = 37), and 20 (*n* = 36) μg/ml brivaracetam for 24 h in physiological M5 and incubation with 0.5 (*n* = 19), 2 (*n* = 23), 10 (*n* = 22), and 20 (*n* = 25) μg/ml brivaracetam for 24 h in pathological M30 co-cultures and resulted in no significant changes **(A)**. The panels in **(B)** show exemplary anti-Cx43 and DAPI stained cells of M5 and M30 co-cultures incubated with 20 μg/ml brivaracetam **(B-2 and B-4)** and the corresponding control (untreated cells; **B-1 and B-3**). Comparisons between the groups were analyzed using D’Agostino-Pearson normality test and One-way analysis of variance (One-way ANOVA) followed by Bonferroni *post hoc* comparison test. Control: untreated cells; Vehicle: cells incubated with the vehicle 0.9% NaCl diluted in distilled H_2_O (at a ratio of 1:1; 2 μl per ml cell culture medium).

Cx43 signal detected by immunocytochemistry was not affected by incubation of physiological M5 and pathological M30 co-cultures with different concentrations of BRV for 24 h compared to untreated controls [Figure 5A; e.g., from mean 12.68 ± 0.78 signals per cell in control (*n* = 34) to 12.65 ± 0.87 signals per cell after incubation with 20 μg/ml BRV (*n* = 36) in M5 co-cultures (Figures 5A,B-1,B-2); from mean 15.03 ± 0.85 signals per cell in control (*n* = 26) to 12.25 ± 0.95 signals per cell after incubation with 20 μg/ml BRV (*n* = 25) in M30 co-cultures (Figures 5A,B-3,B-4)].

**Figure 6:**
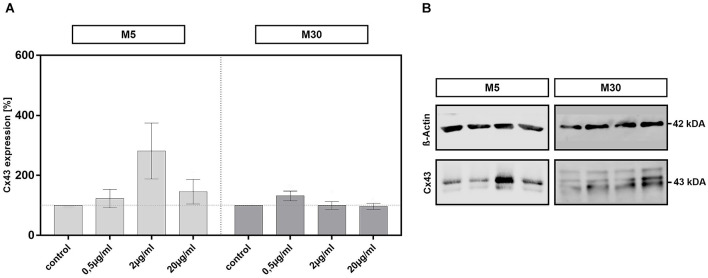
Expression of all forms of Connexin 43 (Cx43) was detected by Western blot analysis after concentration-dependent incubation with 0.5, 2, and 20 μg/ml brivaracetam (*n* = 5 for each concentration) for 24 h in physiological M5 and pathological M30 co-cultures and resulted in no significant changes **(A)**. The panels in **(B)** show exemplary Cx43 western blots of M5 and M30 co-cultures with the corresponding ß-actin as a control for loading (see additional file for original, unprocessed versions of full-length representative western blots). Comparisons between the groups were analyzed using D’Agostino-Pearson normality test and One-way analysis of variance (One-way ANOVA) followed by Bonferroni *post hoc* comparison test. Control: untreated cells.

Incubation of physiological M5 and pathological M30 co-cultures with different concentrations of BRV for 24 h and detection of Cx43 protein in cell lysates by Western blot resulted in no significant changes of Cx43 protein levels compared to untreated controls (*n* = 5 for each experiment; e.g., from an assumed mean Cx43 expression level of 100% in the controls to 145.8 ± 41.14 at 20 μg/ml BRV in M5 co-cultures and to 96.8% ± 10.73 at 20 μg/ml BRV in M30 co-cultures; Figures 6A,B).

### Effects of BRV on cell-cell communication in astrocyte-microglia co-cultures

**Figure 7:**
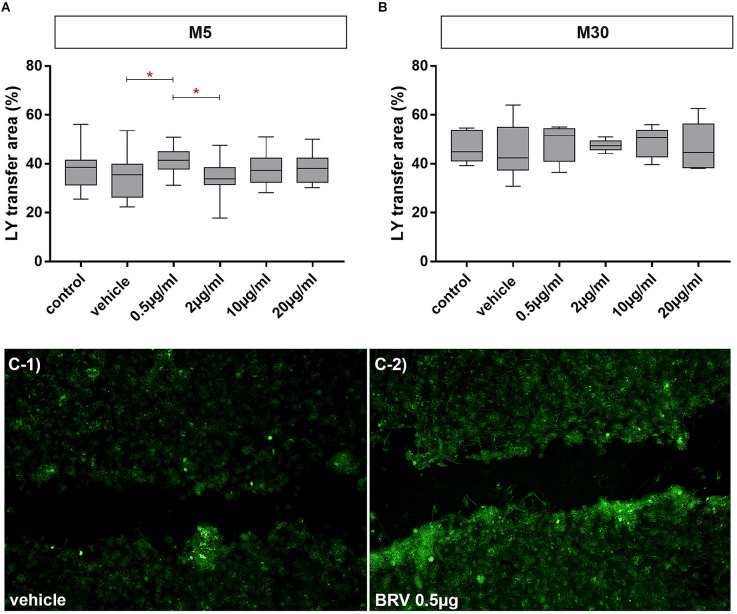
Effects on gap-junctional cell-cell communication in M5 and M30 co-cultures after incubation with different concentrations of brivaracetam for 24 h (examined by scrape loading method). In the physiological M5 co-cultures, incubation with 0.5 μg/ml brivaracetam (*n* = 20) led to a slight significant increase in the cell-cell communication **(A)**. In the pathological M30 co-cultures, incubation with brivaracetam for 24 h led to no significant changes in intercellular communication **(B)**. The panels **(C-1/C-2)** show an example fluorescence micrograph [Lucifer Yellow (LY) marked cells] of the control with vehicle **(C-1)** compared to incubation with 0.5 μg/ml brivaracetam **(C-2)** of the physiological M5 co-culture. Comparisons between the groups were analyzed using D’Agostino-Pearson normality test and One-way analysis of variance (One-way ANOVA) followed by Bonferroni *post hoc* comparison test. Differences were considered significant at *p* < 0.05: *. Control: untreated cells; Vehicle: cells incubated with the vehicle 0.9% NaCl diluted in distilled H_2_O (at a ratio of 1:1; 2 μl per ml cell culture medium).

Incubation of physiological M5 co-cultures with 0.5 μg/ml BRV for 24 h resulted in a slightly significant increase of gap-junctional cell-cell communication (increase of Lucifer Yellow stained cells to 41.45% ± 1.17 (*n* = 20; *p* < 0.05: *; Figures 7A,C-1,C-2). The cell-cell communication was unchanged under all conditions in M30 co-cultures (Figure 7B).

## Discussion

In this study, the glial cell viability was significantly reduced after incubation with a high, overdose concentration (20 μg/ml) of BRV compared to controls and lower, therapeutic concentrations (0.5 and 2 μg/ml) under physiological conditions. However, this effect was not observed after a concentration-dependent incubation with BRV under pathological, inflammatory conditions. Astroglial Cx43 expression detected by immunocytochemistry and Western blot was not affected under all conditions. The functional coupling *via* GJs was not changed under inflammatory conditions. Interestingly, incubation with a low therapeutic concentration (0.5 μg/ml) of BRV led to a slightly significant increase in GJ coupling under physiological conditions. Moreover, incubation with therapeutic concentrations of BRV (0.5 and 2 μg/ml) led to a significant reduction of resting microglial phenotype and a significant increase of activated microglial phenotype under inflammatory conditions.

Previous data support a link between neuroinflammation and epilepsy (Vezzani et al., 2011; Eyo et al., 2014, 2017; Sofroniew, 2014; Aronica et al., 2017; French et al., 2017; Patel et al., 2019). Inflammatory molecules have been found in experimental models of epilepsy and in surgically resected human brain tissue from patients with treatment-resistant epilepsy (Aronica et al., 2017). ASMs can affect cell-mediated immunity but involved mechanisms are not well understood (Beghi and Shorvon, 2011). The epileptic activity was associated with microglial activation (Beach et al., 1995; Boer et al., 2006; Eyo et al., 2014). Immunoreactive microglial cells were increased in hippocampal sclerosis associated with human temporal lobe epilepsy, compared to control autopsy hippocampus (Beach et al., 1995). Moreover, a specific and persistent increase in the density of activated microglia correlated with the duration of epilepsy and frequency of seizures in human focal cortical dysplasia, supporting the contribution of the inflammatory processes to epileptogenicity (Boer et al., 2006). In addition to human studies, morphological changes of microglia with an increased number of microglial branch numbers were found in the hippocampus during kainic acid (KA)-induced seizure activity in mice (Eyo et al., 2014). Glutamate is released from neurons during periods of intense neuronal hyperactivity as occurring in epilepsy-activated neuronal NMDA receptors, resulting in calcium influx and ATP release. This induced microglial process extension toward neuronal elements, indicating the existence of neuron-to-microglia communication (Eyo et al., 2014).

Our previous studies revealed different effects of ASMs on astrocytes and microglia (Haghikia et al., 2008; Stienen et al., 2011; Dambach et al., 2014; Ismail et al., 2021; Corvace et al., 2022; Faustmann et al., 2022). For example, PHE, GBT, and LTG did not influence the microglial phenotypes in our astrocyte-microglia co-culture model (Dambach et al., 2014; Faustmann et al., 2022). Strong microglial activation was induced by VPA under physiological and pathological conditions (Dambach et al., 2014). Anti-inflammatory glial features with inhibition of microglial activation were demonstrated after incubation with CBZ, TPM, and LCM under inflammatory conditions (Dambach et al., 2014; Corvace et al., 2022; Faustmann et al., 2022). Furthermore, LEV decreased the enhanced pro-inflammatory interleukin-1β (IL-1β) level and promoted the expression of anti-inflammatory transforming growth factor-β1 (TGF-β1) under inflammatory conditions, suggesting anti-inflammatory features in our co-culture model (Haghikia et al., 2008; Stienen et al., 2011). Although BRV has high brain permeability, its effect on neuroinflammation with regard to astrocytes and microglia has not yet been completely explored and only a few studies are available on this topic. Previous findings showed that LEV and BRV together were associated with a significantly increased risk of infection, suggesting pro-inflammatory features (Zaccara et al., 2017). Inhibitory effects on CD8^+^ T lymphocytes induced by LEV were demonstrated and related to increased incidence of upper respiratory tract infections in LEV-treated patients (Li et al., 2013). Because SV2A protein is also expressed in human CD8^+^ T lymphocytes, inhibition of SV2A function was proposed to be responsible for this effect (Li et al., 2013). Otherwise, in a murine model of neuropathic pain, BRV significantly reduced microglial activation and leukocyte infiltration into the dorsal horn of the spinal cord that was related to reduced neuropathic pain behaviors (Tsymbalyuk et al., 2019). Interestingly, BRV in therapeutic concentrations (0.5 and 2 μg/ml) increased the microglial activation in our inflammatory astrocyte-microglia co-culture model. The inactivated resting microglia were also decreased by BRV (2 and 10 μg/ml) in our physiological co-cultures. Our findings suggest mild pro-inflammatory, *in vitro* features of BRV with regard to microglia. Future studies with investigation of further microglial activation markers and signaling pathways after treatment with BRV could be useful to understand the underlying mechanisms. We expect that LEV may exert both pro- and anti-inflammatory features depending on the context because the level of pro-inflammatory cytokines was decreased by LEV and suppression of inflammatory reactions in the mouse microglial cell line BV-2 was demonstrated (Haghikia et al., 2008; Stienen et al., 2011; Niidome et al., 2021). On the other hand, LEV may trigger infections (Li et al., 2013; Zaccara et al., 2017).

The proteins SV2A and SV2B regulate presynaptic Ca^2+^ accumulation, action potential-dependent neurotransmitter release in e.g., hippocampal neurons and their absence induces epilepsy e.g., SV2A deficiency showed elevated seizure vulnerability in an SV2A knockout model of zebrafish (Janz et al., 1999; Sills, 2010; Zhang et al., 2022). LEV and BRV seem to induce or stabilize different conformations of the SV2A protein (Klitgaard et al., 2016). It was reported that in an inflammatory mouse model of neuropsychiatry systemic lupus erythematosus microglial cells are activated and showed an elevated uptake of synaptic material *via* staining of intracellular SV2A protein compared to wild type microglia (Makinde et al., 2020). Microglial-dependent synapses loss in CNS lupus was here type I Interferon mediated (Bialas et al., 2017) and could contribute to epilepsy (Andoh et al., 2019). These findings are highly relevant since they describe another mechanism involving SV2A and microglia in a different inflammatory CNS model and possible association with epilepsy. On the other hand, LEV seems to suppress inflammatory reactions in the mouse microglial cell line BV-2 which did not express SV2A (Niidome et al., 2021). It is known that the anticonvulsive effect of BRV is mediated by SV2A but it remains unclear if the microglial pro-inflammatory effect of BRV in our co-cultures is also SV2A-mediated.

The dose-dependent cytotoxic and anti-migratory effects of BRV on human glioma cells were previously described (Rizzo et al., 2017). Incubation of glioma cells with BRV resulted in the modulation of several microRNAs e.g., miR-195-5p modulation which affects the cell cycle (Rizzo et al., 2017). Previous studies about the toxicity of BRV on neuronal or glial cell cultures are not available. In our study, BRV has not shown effects on glial cell viability under inflammatory conditions. However, reduced glial cell viability was detected after treatment of physiological co-cultures with high concentration, suggesting toxic effects on glial cells by overdose.

There is a strong evidence based on epileptic animal models and human epilepsy studies that glial Cx-based GJs and hemichannels are involved in epilepsy (Szente et al., 2002; Condorelli et al., 2003; Samoilova et al., 2003; Mylvaganam et al., 2010, 2014). Increased astrocytic Cx43 expression and inter-glial GJ communication were associated with epileptiform activity (Mylvaganam et al., 2010, 2014). There is a previous study investigating the BRV effects on Cx43 (Okada et al., 2021). A subchronic administration of BRV inhibited the activation of astroglial L-glutamate release as well as the expression of SV2A and Cx43 in the plasma membrane of rat primary cultured astrocytes, but these effects were not detected after the acute administration of BRV (Okada et al., 2021). It has been suggested that the migration of SV2A to the astroglial plasma membrane by hyperexcitability leads to the activation of astroglial glutamatergic transmission *via* hemichannel activation (Okada et al., 2021). Further, Fukuyama and Okada (2022) electrophysiologically investigated primary cultured rat astrocytes using high-frequency oscillation (HFO). Here, subchronic administration of BRV and LEV decreased fast-ripple HFO-induced astroglial L-glutamate release and expression of Cx43 and SV2A in the plasma membrane. The decrease of Cx43 expression was not found for BRV treatment when using subchronic ripple HFO-evoked stimulation (Fukuyama and Okada, 2022). The antiseizure effect of BRV was discussed to be mediated by suppression of SV2A and subsequent inhibition of turnover prolongation of Cx43 hemichannels in the plasma membrane (Fukuyama and Okada, 2022). In our co-culture model, astroglial Cx43 expression detected by immunocytochemistry and Western blot was not significantly affected after treatment with BRV. The functional coupling *via* GJs was not changed under inflammatory conditions, whereas incubation with a low therapeutic concentration of BRV increased slightly the GJ coupling under physiological conditions. The relevance of these minimal changes should be viewed with caution. It has been previously demonstrated that microglial activation correlated with reduced astroglial Cx43 expression under inflammatory *in vitro* conditions (Faustmann et al., 2003). After incubation with therapeutic concentrations of BRV, the microglial activation was slightly increased in our inflammatory astrocyte-microglia co-culture model, but without modulation of Cx43 expression. It is possible that longer incubation (>24 h) with BRV could lead to changes in Cx43 expression. Furthermore, the modification of all forms of Cx43 (panCx43) was investigated in our study, but without identification of the phosphorylation status. There is evidence that phosphorylation regulates both the structure and function of Cx43, contributing to changes in localization, interacting protein partners, channel selectivity, and intercellular communication (Solan and Lampe, 2009). In contrast to our study, Okada et al. (2021) and Fukuyama and Okada (2022), on the one hand, described subchronic BRV treatment which reduced Cx43 and SV2A expression, but not for acute BRV treatment and, on the other hand, subchronic electrophysiological stimulation with no influence on Cx43 expression by BRV, but for fast electrophysiological stimulation (decrease of Cx43 and SV2A), suggesting different effects of BRV by different ways of substance administration and different ways of stimulation in astroglial cultures. We can conclude that another point of influence could be the presence of microglia in our astrocyte-microglia co-culture model, leading to a slight increase of GJ coupling under physiological conditions but the relevance of these minimal changes is not clear. Moreover, BRV concentrations (0.1 and 3 μm used by Fukuyama and Okada, 2022; 1.3 and 10 μm used by Okada et al., 2021) vary from our BRV concentrations and also the use of astrocytes monocultures could lead to different results as well. Western Blot-detection of SV2A protein in our astrocyte-microglia co-culture model would be interesting for future experiments. Additional electrophysiological experiments in our co-cultures are necessary to confirm the effects of BRV on GJ channels and research about the effects of BRV on Cx43 phosphorylation status may help to explain the underlying mechanisms.

The lack of investigations on inflammatory cytokines such as tumor necrosis factor (TNF)-α, IL-1β, or IL-6 after incubation with BRV is one of the limitations of our study. Additional studies with investigation of further microglial activation markers and signaling pathways after treatment with BRV are also necessary. Moreover, the further impact of BRV on neuroinflammation in epileptic animal models is a topic for future research, however, the focus of the current study was on *in vitro* effects of BRV.

In addition, our results in the astrocytes-microglia co-culture model of inflammation could be relevant in a circuit with neuronal populations. Based on previous findings about microglia-neuron interactions, it has been shown that microglia are able to regulate neuronal activities in healthy and epileptic brain (Eyo et al., 2017). Microglial activation at morphological and molecular levels was described during and following seizures. Microglia may attenuate or exacerbate seizure intensity during seizures and neuronal dysfunction following seizures, contributing to seizure-induced neurodegeneration and neurogenesis (Eyo et al., 2017). Thus, we expect that research involving astrocytes, microglia and neurons may confirm similar findings as in our study.

Our astrocyte-microglia co-culture model is an established *in vitro* model in which ASMs with different mechanisms of action such as LCM, LTG, TPM have already been studied. There is a current study about effects of BRV in our co-culture model, offering new insights into this drug.

## Conclusions

The implication of astrocytes and microglia in the pathophysiology of epilepsy raises the question of how these cells besides neurons may be responsive to treatment with ASMs. In this *in vitro* study, we revealed the effects of the ASM BRV on glial cells using the astrocyte-microglia co-culture model of inflammation. Treatment with BRV in therapeutic concentrations increased the microglial activation in our inflammatory astrocyte-microglia co-culture model. The inactivated resting microglia were also decreased by BRV in our physiological co-cultures. These findings suggest mild pro-inflammatory, *in vitro* features of BRV with regard to microglia. BRV did not affect the Cx43 expression and showed only limited effects on gap-junctional coupling. Incubation with a high concentration of BRV reduced the glial cell viability in our physiological co-cultures, suggesting possible toxic effects by overdose.

## Data Availability Statement

The original contributions presented in the study are included in the article/Supplementary material, further inquiries can be directed to the corresponding author.

## Ethics Statement

The use of vertebrate animals for scientific purposes in Germany is regulated by the Animal Welfare Act and it is the duty of the institutional Animal Welfare Officer that animal experiments are carried out in compliance with the regulations of the Animal Welfare Act, and other animal protection regulations applicable within the European Union. According to current German and European legislation, the removal of organs or cells from vertebrates for scientific purposes is not considered an animal experiment if the animals have not been subject to surgical interventions or invasive treatments prior to sacrifice. Thus, euthanization of mice intended for the removal of brain tissue does not need approval or permission from local or governmental authorities. The experimental protocols used in the research project for generating primary cell cultures have been approved as being in accordance with animal protection regulations. Housing and husbandry of the experimental animals that have been used for the research project were in accordance with all applicable legal regulations and that personnel and housing facilities had the necessary official permissions.

## Author Contributions

FI is responsible for concepts and design of the study, performed the experiments and is responsible for data collection, and prepared the manuscript. FC is responsible for statistical analysis. All authors contributed intellectually. All authors acquired, analyzed, and interpreted the data. All authors reviewed and made critical revisions to the manuscript. All authors provide approval for publication of the content. All authors agree to be accountable for all aspects of the work in ensuring that questions related to the accuracy or integrity of any part of the work are appropriately investigated and resolved. All authors contributed to the article and approved the submitted version.

## Funding

This research did not receive any specific grant from funding agencies in the public, commercial, or not-for-profit sectors.

## Supplementary Material

Supplementary Figure S1 Original, unprocessed versions of full-length representative western blots with regard to Figure 6, showing connexin (Cx) 43 protein expression (compared to ß-actin as a control for loading) in M5 **(A)** and M30 **(B)** astrocyte-microglia co-cultures after incubation with different concentrations of brivaracetam for 24 h.
