# Supplementary figures and images for: Brivaracetam exhibits mild pro-inflammatory features in an in vitro astrocyte-microglia co-culture model of inflammation
Source: Front Cell Neurosci. 2022 Nov 3;16:995861. doi: 10.3389/fncel.2022.995861 (PMC9670320; doi:10.3389/fncel.2022.995861)

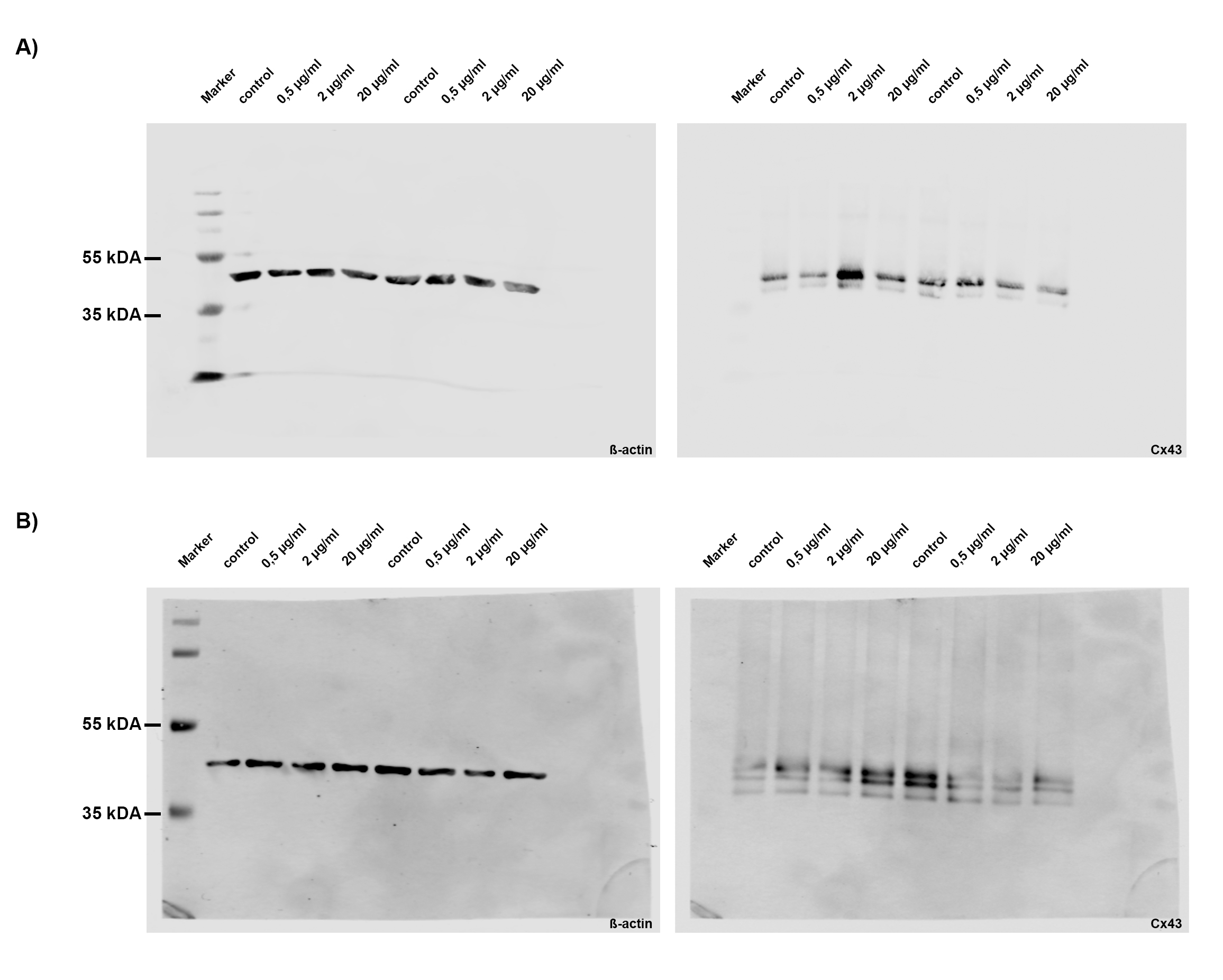

Supplement: Supplementary Figure S1 — Original, unprocessed versions of full-length representative western blots with regard to Figure 6, showing connexin (Cx) 43 protein expression (compared to ß-actin as a control for loading) in M5 (A) and M30 (B) astrocyte-microglia co-cultures after incubation with different concentrations of brivaracetam for 24 h. [file Image_1.JPEG]
